# Long‐term fasting glucose variability and risk of cancer in patients with type 2 diabetes mellitus: A retrospective population‐based cohort study in Shanghai

**DOI:** 10.1111/1753-0407.13329

**Published:** 2022-11-09

**Authors:** Xiao‐rui Cui, Jun Li, Ya‐ting Yang, Jing‐yi Wu, Hui‐lin Xu, Yong‐fu Yu, Guo‐you Qin

**Affiliations:** ^1^ Department of Biostatistics, School of Public Health, and The Key Laboratory of Public Health Safety of Ministry of Education Fudan University Shanghai China; ^2^ Shanghai Minhang Center for Disease Control and Prevention Shanghai China; ^3^ Shanghai Institute of Infectious Disease and Biosecurity Shanghai China

**Keywords:** cancer, fasting glucose variability, hypertension, interaction, type 2 diabetes mellitus, 癌症, 空腹血糖变异性, 高血压, 2型糖尿病互作。

## Abstract

**Backgrounds:**

Fasting blood glucose (FBG) variability may make an impact on adverse events in patients with diabetes mellitus. However, the association between long‐term changes in FBG and cancer remains unclear. We aimed to investigate this association in a large‐scale longitudinal study.

**Methods:**

Data were collected from 46 761 patients with type 2 diabetes mellitus aged 20–80 years who participated in the Diabetes Standardized Management Program in Shanghai, China. We adopted four indicators, including standard deviation (SD), coefficient of variation (CV), variation independent of the mean (VIM), and average real variability (ARV) to describe FBG variability. Adjusted multivariable Cox regression analyses and restricted cubic splines were used to investigate the association between long‐term FBG variability and cancer risk. We also determined the interactive effect of FBG variability with hypertension and FBG‐mean with hypertension on cancer risk, respectively.

**Results:**

In this study, we confirmed 2218 cancer cases (51.1% male) over a median follow‐up of 2.86 years. In the multivariable‐adjusted models, participants in the highest quartile of FBG variability had an increased risk of cancer compared with those in the lowest quartile. The nonlinear association was found when using FBG‐VIM, FBG‐ARV, and FBG‐SD in restricted cubic spline plots. There was a significant interaction effect of FBG variability with hypertension on cancer, whereas the effect of FBG‐mean with hypertension did not attain significance.

**Conclusions:**

Our retrospective cohort study demonstrated a positive association between the long‐term changes in FBG and cancer risk in patients with type 2 diabetes mellitus. FBG variability may independently predict cancer incidence.

## Graphical abstract

Highlights

We identified a positive and nonlinear association between FBG variability and cancer risk in patients with T2DM patients. An interaction effect between FBG variability and hypertension on cancer incidence was identified.

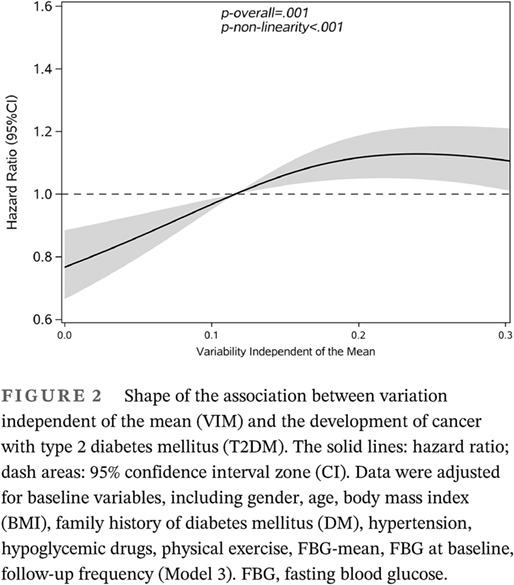

## 摘要

**背景:**

空腹血糖(FBG)变异性可能对糖尿病患者的不良事件产生影响。然而，空腹血糖的长期变化与癌症之间的联系仍不清楚。本研究旨在一项大规模纵向研究中调查和量化这种关联。

**方法:**

对参加中国上海市糖尿病标准化管理项目的46,761例年龄在20到80岁的2型糖尿病患者进行调查。我们采用标准差(SD)、变异系数(CV)、独立于均值的变异(VIM)和平均实际变异(ARV)4个指标来描述FBG的变异性。调整后的多变量COX回归分析和限制三次样条法被用来调查长期空腹血糖(FBG)变异性与癌症风险之间的关系。我们还分别确定了空腹血糖变异性与高血压和空腹血糖均值与高血压之间的交互作用对癌症风险的影响。

**结果:**

在这项研究中，我们确认了2218例癌症病例(51.1%为男性)，平均随访时间为2.86年。在多变量校正模型中，FBG变异性最高四分位的参与者比那些最低四分位数的参与者患癌症的风险更高。在限制三次样条图中使用FBG‐VIM、FBG‐ARV和FBG‐SD时，发现了非线性关联。FBG变异性与高血压之间存在显著的交互作用，而FBG均值与高血压之间的交互作用不显著。

**结论:**

我们的回顾性队列研究显示，FBG的长期变化与2型糖尿病患者的癌症风险呈正相关。FBG变异性可独立预测癌症发病率。

## 1 INTRODUCTION

China^1^ has the largest number of diabetes patients globally, most of whom havetype 2 diabetes mellitus (T2DM), the fastest developing metabolic disorders and functional problems.^2^, ^3^, ^4^, ^5^ Without proper management, T2DM could result in detrimental complications, including cancer. Several large‐scale epidemiologic and biological studies have shown a consistent rise in cancer incidences among patients with T2DM.^6^, ^7^ It is widely reported that fasting glucose level is independently associated with hazards of cancer onset,^8^, ^9^ with potential mechanisms including endothelial injury and hyperinsulinemia status. However,^9^, ^10^ more intensive glucose control to reduce glucose level do not lower the risk of cancer incidence. Apart from fasting glucose level alone, fasting blood glucose (FBG) variability may contribute more to increasing cancer onset due to oxidative stress and inflammation pathways.

Prior studies^11^, ^12^, ^13^ have mainly concentrated on the relationship between FBG variability and specific site of cancers and have been conducted on populations without diabetes mellitus (DM) or a relatively small population.^13^, ^14^ Besides, population‐based studies on the dose–response relationship between FBG variability and cancer in T2DM patients are scarce. Additionally,^15^ hypertension and T2DM seem to be two aspects of a common physiological mechanism,^16^ and a vast population of T2DM is also affected by hypertension.^17^ Shared risk factors such as hypertension could affect both T2DM and cancer, such as exercise, diet, etc. Thus, more studies are necessary to reveal the association between FBG variability and all‐sites cancer within T2DM patients using various measurements, as well as the role of comorbidity with hypertension in this process.

In this cohort study, we sought to estimate the correlation between long‐term FBG variability and cancer incidence among T2DM patients. Beyond that, the secondary objective was to examine the interaction of FBG variability with hypertension and FBG‐mean with hypertension on cancer risk.

## 2 MATERIALS AND METHODS

### 2.1 Study population

In this retrospective cohort study, participants were drawn from the Diabetes Standardized Management Program (DSMP) in Shanghai's Minhang District, China, including more than 50 000 Chinese patients who had been given a T2DM diagnosis based on the World Health Organization diagnostic criteria from 1999. The DMSP was launched as a basic public health service program from 2004 in Minhang District and covered over 1 million residents in Shanghai.^18^, ^19^ More detailed information can be found in previous literature. A total of 51 970 patients aged between 20 and 80 years were initially included in the study from 2004 to 2015. They were regularly followed up by general practitioners (GPs) during this period and all data were collected in electronic health records (eHRs).

**FIGURE 1:**
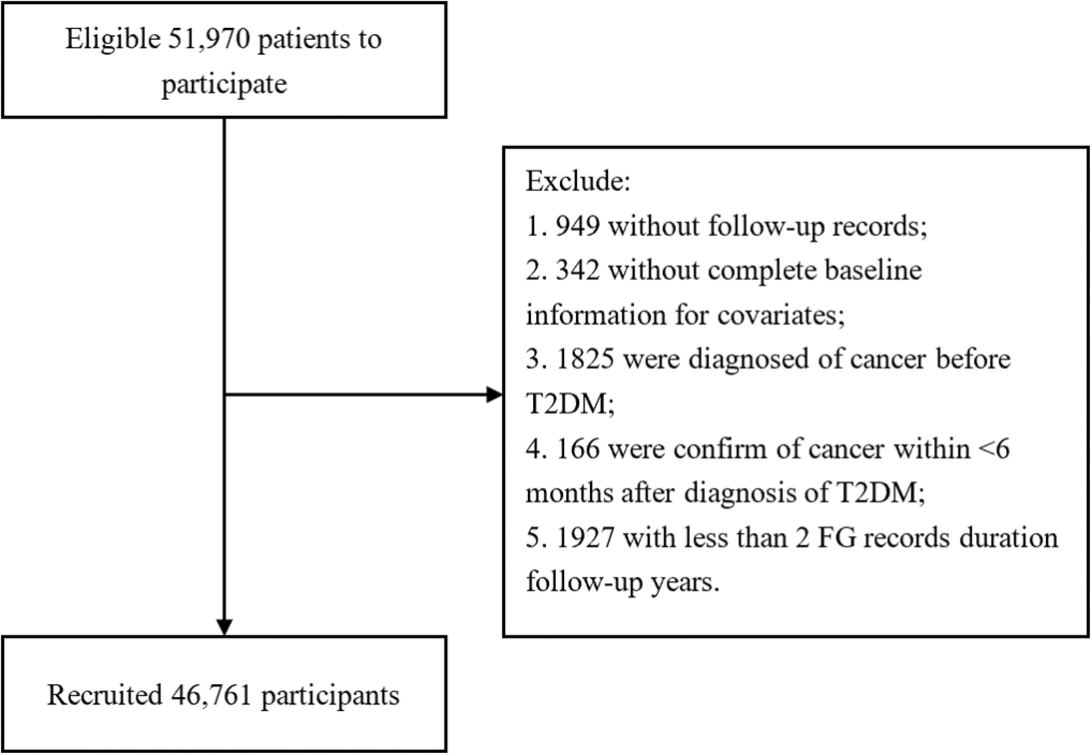
Flow chart for study participant filtration. FG, fasting glucose; T2DM, type 2 diabetes mellitus.

Participants were excluded for the following reasons: lack of FBG record, missing baseline data for exposure to any necessary covariates, past or present history of cancer at baseline, less than 6 months between diagnosis of cancer and T2DM, or < 2 FBG measurements during the course. In the end, 46 761 participants were enrolled in the cohort study (Figure 1).

Ethical approval was unnecessary due to the use of encrypted retrospective information in eHRs.

### 2.2 Data collection

Participants were interviewed by GPs at least four times a year with standardized questionnaires to collect gender, age, daily exercise, and frequency of follow‐up. Patients self‐reported their weight, height, hypoglycemic drug, and family history of diabetes. Body mass index (BMI) was calculated as body weight in kilograms divided by the square of height in meters. We also matched with the database of hypertensive patients in the community to confirm the comorbidity of hypertension.

FBG variability was defined by four indicators: standard deviation (SD), coefficient of variation (CV), variation independent of the mean (VIM), and average real variability (ARV). CV was calculated as the SD/mean × 100%; VIM as 100 * SD/mean^*β*^, where *β* is the regression coefficient based on the natural logarithm of SD on the natural logarithm of the mean; and ARV as the average of the absolute differences between consecutive glucose measurements according to the following formula, where *n* refers to the number of records of FBG. A total of 988 641 follow‐up records ranging from 3 to 20 mmol/L of FBG measurement were selected for this study. After the exclusion, the FBG of each individual was measured 2 to 100 times (median [interquartile range]: 9 [18, 29]).^18^

$$\mathrm{ARV}=\frac{1}{n-1}\sum_{i=1}^{n-1}\left|{\mathrm{FG}}_{i+1}-{\mathrm{FG}}_{i}\right|$$

### 2.3 Definition of outcomes

The end point of this study was the incidence of cancer cases. Cancer events (Classification of Diseases and Related Health Problems, 10th Revision, Clinical Modification, C00‐C97) were ascertained based on the Shanghai Cancer Registry through a unique ID card number. The Shanghai Cancer Registry was established as a population‐based cancer registry to gather and analyze data on cancer incidence and mortality among Shanghai residents since 1963.^20^ It managed and received all newly diagnosed cancer cases from more than 160 medical facilities in Shanghai, hence its reliability could be confirmed. Follow‐up began on the date of registration for DSMP and ended at the earliest incidence of cancer or end of the study (December 31, 2015).

### 2.4 Statistical analysis

Participants were grouped by whether cancer occurred. Baseline characteristics were presented as median (interquartile range) for continuous variables and as frequency (%) for categorical ones; differences in baseline characteristics were assessed using Wilcoxon signed‐rank tests for continuous variables and *χ* ^2^ tests for categorical ones. Additionally, FBG variability indexes were categorized into quartiles and the comparison between groups was analyzed by Kruskal–Wallis tests. The crude incidence rate for cancers was calculated by the number of newly diagnosed cancer cases divided by the number of observed person‐years.

Subsequently,^21^ we assessed the proportional hazards (PH) assumption by examining Schoenfeld residuals and log(−log) survival plots.^22^ According to other studies, if the PH assumption was not met, the interaction would be added to the model as a time‐dependent covariate. In addition, multiple linear regression was used to calculate the variance inflation factor (VIF) to check the collinearity of variables. If VIF with variable was <10, the collinearity assumption was satisfied.

Cox proportional hazard models were used to estimate the hazard ratios (HRs) and 95% confidence intervals (CIs) of the association between FBG variability and cancer risk. FBG variability and other potential covariates were set as the dummy variables in the models and the reference group was the lowest quartile for variability. Three models were run: an unadjusted model (Model 1), a semi‐adjusted model that included gender and age (Model 2), and a fully adjusted model that included all covariates (Model 3).

We further used restricted cubic splines with three knots located on the 5th, 50th, and 95th percentiles to flexibly model the underlying nonlinearity relationship between FBG variability and cancer incidence.^23^ The 25th FBG variability was chosen as the reference. A *p* value of nonlinearity <.05 suggested a nonlinear association between exposure and cancer.

We measured the interactive effects of hypertension with FBG variability and hypertension with FBG‐mean on the multiplication and additive scale, respectively.^24^ According to the recommended glycemic control goals by Standards of Medical Care in Diabetes from the American Diabetes Association, the FBG‐mean was grouped by 7.2 mmol/L. Each FBG variability indicator was categorized into two groups by the median values in interactive effect analysis. Multiplicative interaction was tested by adding a product term (ie, hypertension × FBG variability and hypertension × FBG‐mean) to Model 3. Additive interaction was assessed by relative excess risk (RERI) due to interaction, which evaluated whether the risk due to dual exposure is greater than the sum of the risks due to each condition. RERI was calculated by the formula as follows: $\text{RERI}={\mathrm{HR}}_{11}-{\mathrm{HR}}_{10}-{\mathrm{HR}}_{01}+1$. When RERI was positive, it indicated significantly increased cancer risk due to the dual exposure (null hypothesis: RERI = 0).

SAS version 9.4 software (SAS Institute, Cary, NC, USA) was used to analyze the data. All statistical analyses were the two‐sided test, and *p* < .05 was considered statistically significant.

### 2.5 Data and Resource Availability

The data sets generated and analyzed during the current study are available from the corresponding author upon reasonable request.

## 3 RESULTS

### 3.1 General condition of the study population

A total of 46  761 participants were included, and 2218 of them (4.7%) developed all‐sites cancer throughout a median follow‐up of 2.86 years. In the total population, the median age was 62.5 years and 51.1% were men. The median follow‐up period was 2.9 (1.4, 4.9) years for individuals with cancer and 3.8 (2.9, 7.5) years for those without cancer. The detailed traits of the subjects are displayed in Table 1. We found statistically significant differences between gender, age, family history of DM, comorbidity of hypertension, and hypoglycemic drug use (*p* < 0.05) except BMI, FBG‐mean, FBG at baseline, and follow‐up frequency in the two groups. In the cancer group compared to the noncancer group, there were more male participants (51.1% vs. 46.9%), as well as more hypertensive individuals (77.5% vs. 71.3%). Compared with the noncancer group, the cancer group had a markedly higher baseline age (62.3 vs. 67.4), FBG‐VIM (0.18 vs. 0.16), FBG‐ARV (0.65 vs. 0.60), FBG‐CV (12.22 vs. 11.42), and FBG‐SD (0.82 vs. 0.76).

**TABLE 1:** : Baseline characteristics and lifestyle variables in type 2 diabetes mellitus patients

Variable	Diabetes patients		*p*
	Without cancer (*N* = 44 543)	With cancer (*N* = 2218)	
Gender			<.001
Men	20 885 (46.9)	1133 (51.1)	
Women	23 658 (53.1)	1085 (48.9)	
Age (years), median (IQR)	62.3 (55.8, 70.0)	67.4 (60.0, 74.9)	<.001
<50	4567 (10.3)	108 (4.9)	
50–59	13 228 (29.7)	451 (20.3)	
60–69	15 576 (35.0)	726 (32.7)	
≥70	11 172 (25.1)	933 (42.1)	
BMI (kg/m^2^) median (IQR)	24.2 (22.4, 26.4)	24.2 (22.2, 26.6)	.315
<18.5	888 (2.0)	56 (2.5)	
18.5–24.9	25 804 (58.0)	1261 (56.9)	
25–29.9	15 576 (35.0)	785 (35.4)	
≥30	2275 (5.1)	116 (5.2)	
Family history of DM			<.001
Yes	10 525 (23.6)	376 (17.0)	
No	28 148 (63.2)	1495 (67.4)	
Unknown	5870 (13.2)	347 (5.6)	
Hypertension			<.001
Yes	31 771 (71.3)	1718 (77.5)	
No	12 722 (28.7)	500 (22.5)	
Hypoglycemic drug use			.026
Never	10 828 (24.3)	572 (25.8)	
Insulin injection	2618 (5.9)	108 (4.9)	
Metformin and Sulfonylureas	4577 (10.3)	224 (10.1)	
Sulfonylureas	13 414 (30.1)	705 (31.8)	
Metformin	6712 (15.1)	332 (15.0)	
Others	6394 (14.4)	277 (12.5)	
Physical exercise			<.001
Yes	27 794 (62.4)	992 (44.7)	
No	12 961 (29.1)	518 (23.4)	
Unknown	3788 (8.5)	708 (31.9)	
Mean‐FBG (mmol/L), median (IQR)	6.77 (6.38, 7.29)	6.74 (6.35, 7.26)	.098
FBG at baseline (mmol/L), median (IQR)	7.20 (6.50, 8.40)	7.18 (6.40, 8.33)	.146
Follow‐up frequency, median (IQR)	18 (9, 29)	17 (9, 28)	.525
VIM			<.001
Quartile 1	11 264 (25.3)	42 7 (19.3)	
Quartile 2	11 178 (25.1)	511 (23.0)	
Quartile 3	11 068 (24.9)	623 (28.1)	
Quartile 4	11 033 (24.8)	657 (29.6)	
ARV			<.001
Quartile 1	11 397 (25.6)	452 (20.4)	
Quartile 2	10 882 (24.4)	521 (23.5)	
Quartile 3	11 265 (25.3)	612 (27.6)	
Quartile 4	10 999 (24.7)	633 (28.5)	
CV			<.001
Quartile 1	11 258 (25.3)	433 (19.5)	
Quartile 2	11 123 (25.0)	569 (25.5)	
Quartile 3	11 094 (24.9)	597 (26.9)	
Quartile 4	11 068 (24.9)	623 (28.0)	
SD			<.001
Quartile 1	11 257 (25.3)	434 (19.6)	
Quartile 2	11 104 (25.1)	586 (26.4)	
Quartile 3	11 094 (24.6)	595 (26.8)	
Quartile 4	11 088 (25.0)	603 (27.2)	

*Note*: Data are *n* (%) unless otherwise indicated; Quartile 1, 2, 3, and 4 for VIM were <0.12 units, 0.12–0.16 units, 0.16–0.23 units and ≥0.23 units, respectively. Quartile 1, 2, 3, and 4 for ARV were <0.40 mmol/L, 0.40–0.60 mmol/L, 0.60–0.90 mmol/L and ≥0.90 mmol/L, respectively. Quartile 1, 2, 3, and 4 for CV were <7.80%, 7.80–11.45%, 11.45–16.91% and ≥16.91%, respectively. Quartile 1, 2, 3, and 4 for SD were <0.51 mmol/L, 0.51–0.77 mmol/L, 0.77–1.19 mmol/L and ≥1.19 mmol/L, respectively.

Abbreviations: IQR, interquartile; ARV, average real variability; BMI, body mass index; CV, coefficient of variation; DM, diabetes mellitus; FBG, fasting blood glucose; IQR, interquartile range; VIM, variation independent of the mean.

### 3.2 Cancer risk according to FBG variability

After testing the PH assumption, hypoglycemic drug use did not meet the assumption and was implemented as the time‐dependent variable. Table 2 shows crude cancer incidence in the total population by quartiles of FBG variability represented as VIM, ARV, CV, and SD. For FBG‐VIM, FBG‐ARV, and FBG‐CV, the crude incidence was highest among the top quartile (107.97, 107.17, and 101.12 per 10 000 person‐years, respectively) except FBG‐SD (98.92 per 10 000 person‐years). VIF values showed a low level of collinearity for all independent variables (VIF < 10.0). The HRs and 95% CIs for the associations of FBG variability with cancer incidence were listed in Table 2. In model 1 (crude model) and model 2 (semi‐adjusted), cancer risk was insignificantly related to FBG variability. Model 3 indicated that the highest quartile of FBG‐VIM, FBG‐ARV, FBG‐CV, and FBG‐SD had a markedly high risk of cancer compared to the lowest quartile by 1.20 (95% CI: 1.06, 1.37), 1.14 (95% CI: 1.00, 1.30), 1.21 (95% CI: 1.05, 1.39) and 1.24 (95% CI: 1.07, 1.45), respectively.

**TABLE 2:** : Hazard ratios and 95% confidence intervals for fasting variability and mean

Studied variable	Case (*N*, %)	Crude incidence (10 000 person‐year)	Hazard ratio (95% CI)			*p* nonlinearity
			Model 1	Model 2	Model 3	
VIM (mmol/L)						<.001
Quartile 1	427 (3.7)	102.43	1 (ref)	1 (ref)	1 (ref)	
Quartile 2	511 (4.4)	89.85	0.87 (0.76, 0.99)	0.91 (0.80, 1.03)	1.07 (0.94, 1.22)	
Quartile 3	623 (5.3)	99.65	0.96 (0.85, 1.08)	1.01 (0.89, 1.14)	1.20 (1.06, 1.37)	
Quartile 4	657 (5.6)	107.97	1.04 (0.92, 1.17)	1.06 (0.94, 1.20)	1.20 (1.06, 1.37)	
ARV (mmol/L)						.007
Quartile 1	452 (3.8)	99.97	1 (ref)	1 (ref)	1 (ref)	
Quartile 2	521 (4.7)	93.50	0.92 (0.81, 1.05)	0.94 (0.83, 1.07)	1.04 (0.91, 1.18)	
Quartile 3	612 (5.2)	98.53	0.97 (0.86, 1.09)	1.00 (0.88, 1.13)	1.11 (0.98, 1.26)	
Quartile 4	633 (5.4)	107.17	1.05 (0.93, 1.18)	1.08 (0.96, 1.22)	1.14 (1.00, 1.30)	
CV (%)						
Quartile 1	433 (3.7)	100.91	1 (ref)	1 (ref)	1 (ref)	.070
Quartile 2	566 (4.8)	99.64	0.98 (0.86, 1.11)	1.01 (0.89, 1.14)	1.15 (1.02, 1.31)	
Quartile 3	597 (5.1)	98.29	0.96 (0.85, 1.09)	1.00 (0.88, 1.13)	1.18 (1.04, 1.34)	
Quartile 4	622 (5.3)	101.12	0.98 (0.87, 1.11)	1.05 (0.93, 1.19)	1.21 (1.05, 1.39)	
SD (mmol/L)						
Quartile 1	434 (3.7)	98.98	1 (ref)	1 (ref)	1 (ref)	.018
Quartile 2	586 (5.0)	103.39	1.04 (0.91, 1.17)	1.06 (0.94, 1.20)	1.20 (1.06, 1.36)	
Quartile 3	595 (5.1)	98.48	0.98 (0.87, 1.11)	1.01 (0.90, 1.15)	1.19 (1.05, 1.36)	
Quartile 4	603 (5.2)	98.92	0.98 (0.87, 1.11)	1.06 (0.94, 1.20)	1.24 (1.07, 1.45)	

*Note*: Model 1: unadjusted. Model 2: adjusted for gender and age. Model 3: adjusted for variables in Model 2 and baseline variables, including BMI, family history of DM, hypertension, hypoglycemic drugs, physical exercise, FBG‐mean, FBG at baseline, and follow‐up frequency.

Abbreviations: ARV, average real variability; BMI, body mass index; CI, confidence interval; CV, coefficient of variation; DM, diabetes mellitus; FBG, fasting blood glucose; Ref, reference; VIM, variation independent of the mean.

**FIGURE 2:**
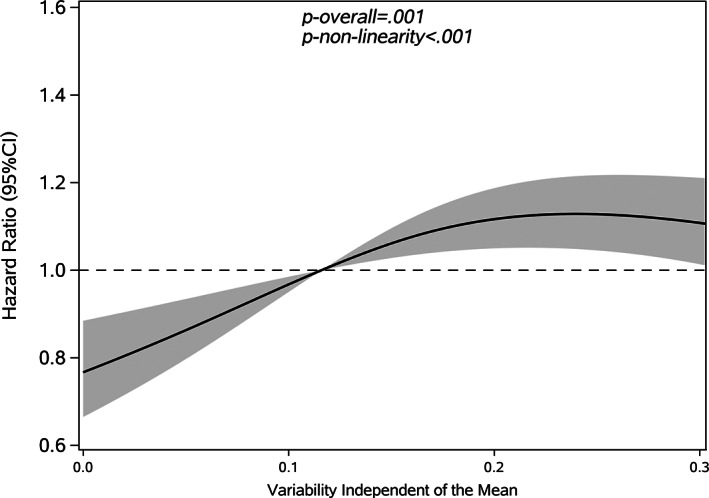
Shape of the association between variation independent of the mean (VIM) and the development of cancer with type 2 diabetes mellitus (T2DM). The solid lines: hazard ratio; dash areas: 95% confidence interval zone (CI). Data were adjusted for baseline variables, including gender, age, body mass index (BMI), family history of diabetes mellitus (DM), hypertension, hypoglycemic drugs, physical exercise, FBG‐mean, FBG at baseline, follow‐up frequency (Model 3). FBG, fasting blood glucose.

**FIGURE 3:**
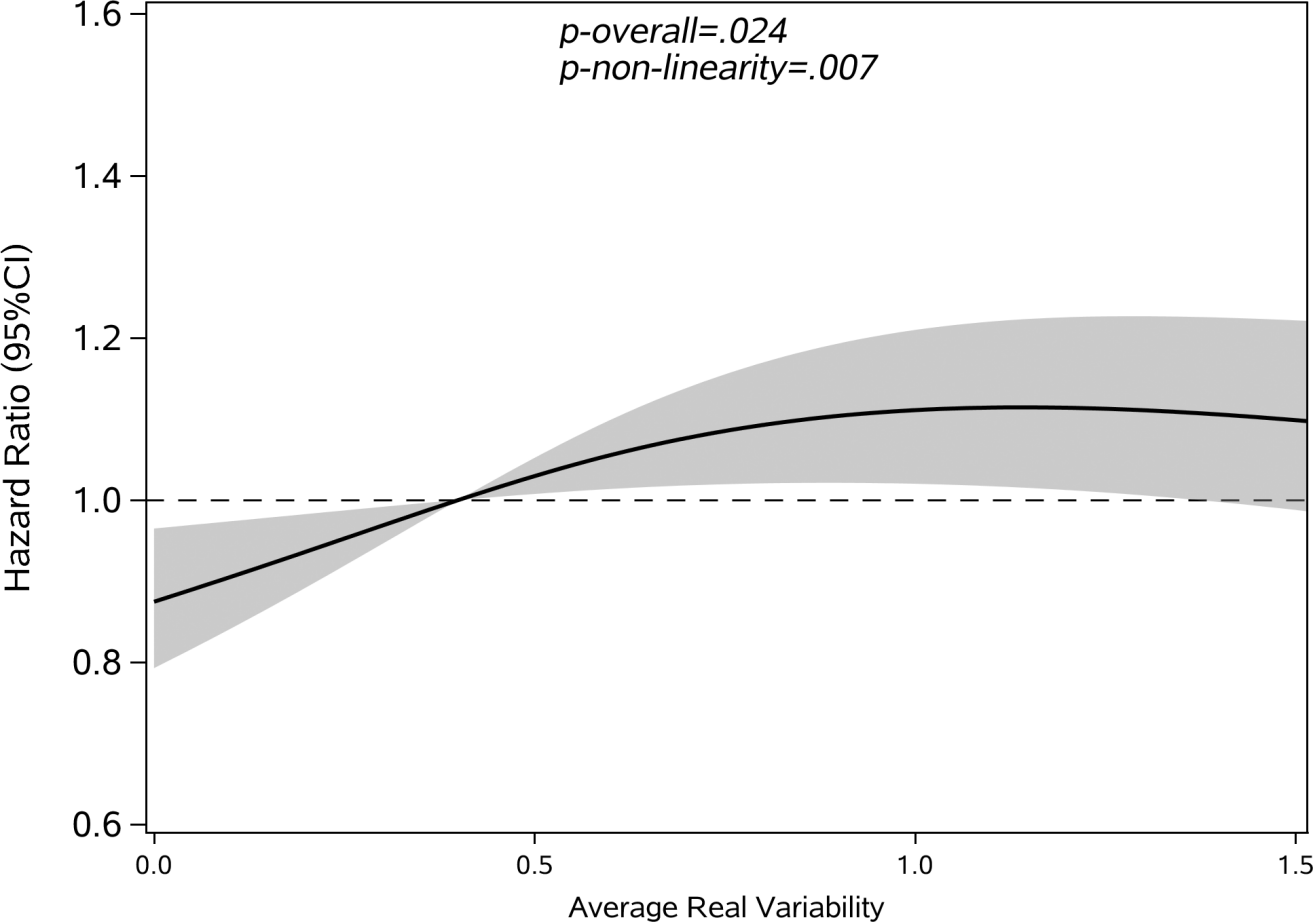
Shape of the association between average real variability (ARV) and the development of cancer with type 2 diabetes mellitus (T2DM). The solid lines: hazard ratio; dash areas: 95% confidence interval zone (CI). Data were adjusted for baseline variables, including gender, age, body mass index (BMI), family history of diabetes mellitus (DM), hypertension, hypoglycemic drugs, physical exercise, FBG‐mean, FBG at baseline, follow‐up frequency (Model 3). FBG, fasting blood glucose.

**FIGURE 4:**
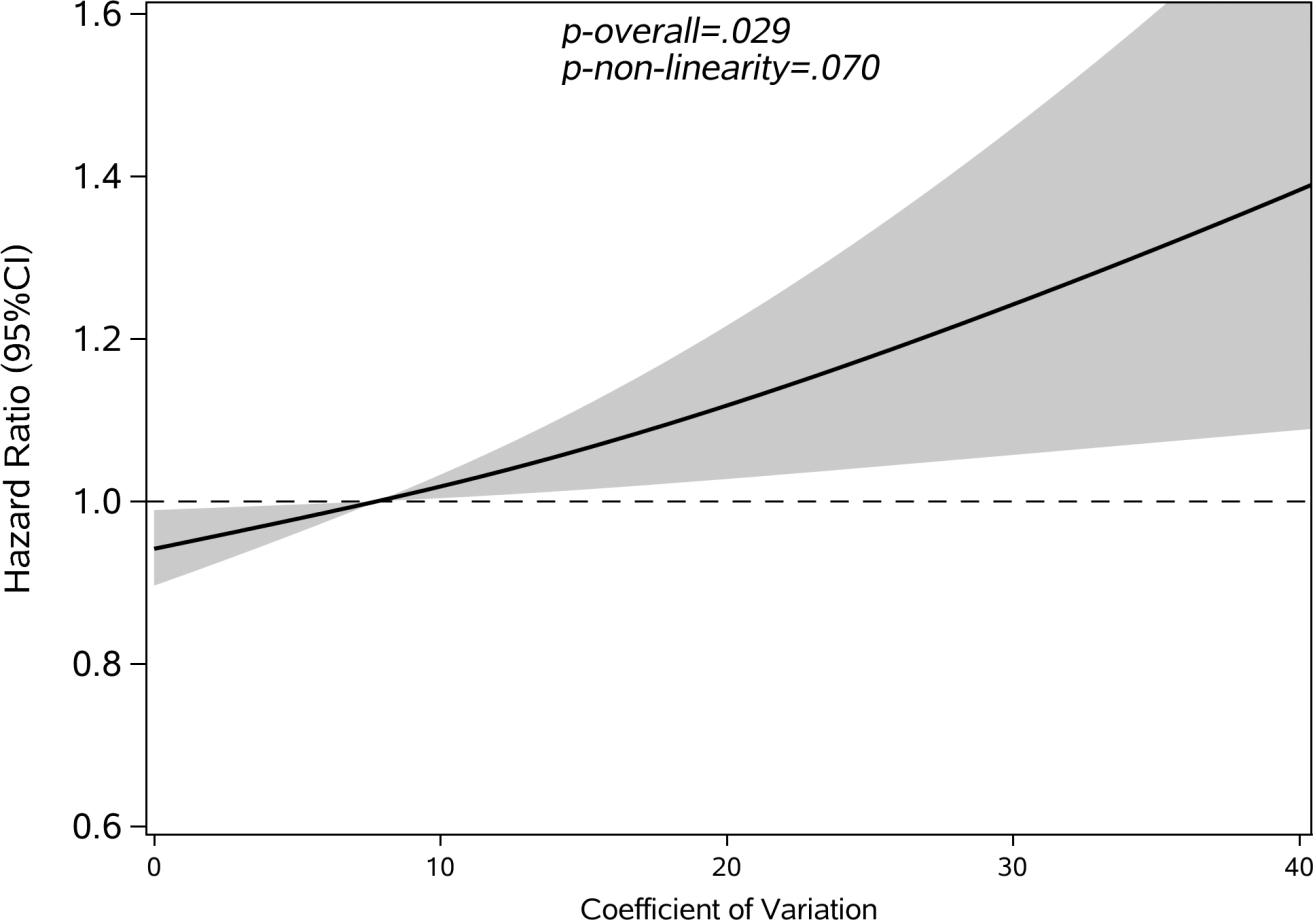
Shape of the association between coefficient of variation (CV) and the development of cancer with type 2 diabetes mellitus (T2DM). The solid lines: hazard ratio; dash areas: 95% confidence interval zone (CI). Data were adjusted for baseline variables, including gender, age, body mass index (BMI), family history of diabetes mellitus (DM), hypertension, hypoglycemic drugs, physical exercise, FBG‐mean, FBG at baseline, follow‐up frequency (Model 3). SD, standard deviation. FBG, fasting blood glucose.

**FIGURE 5:**
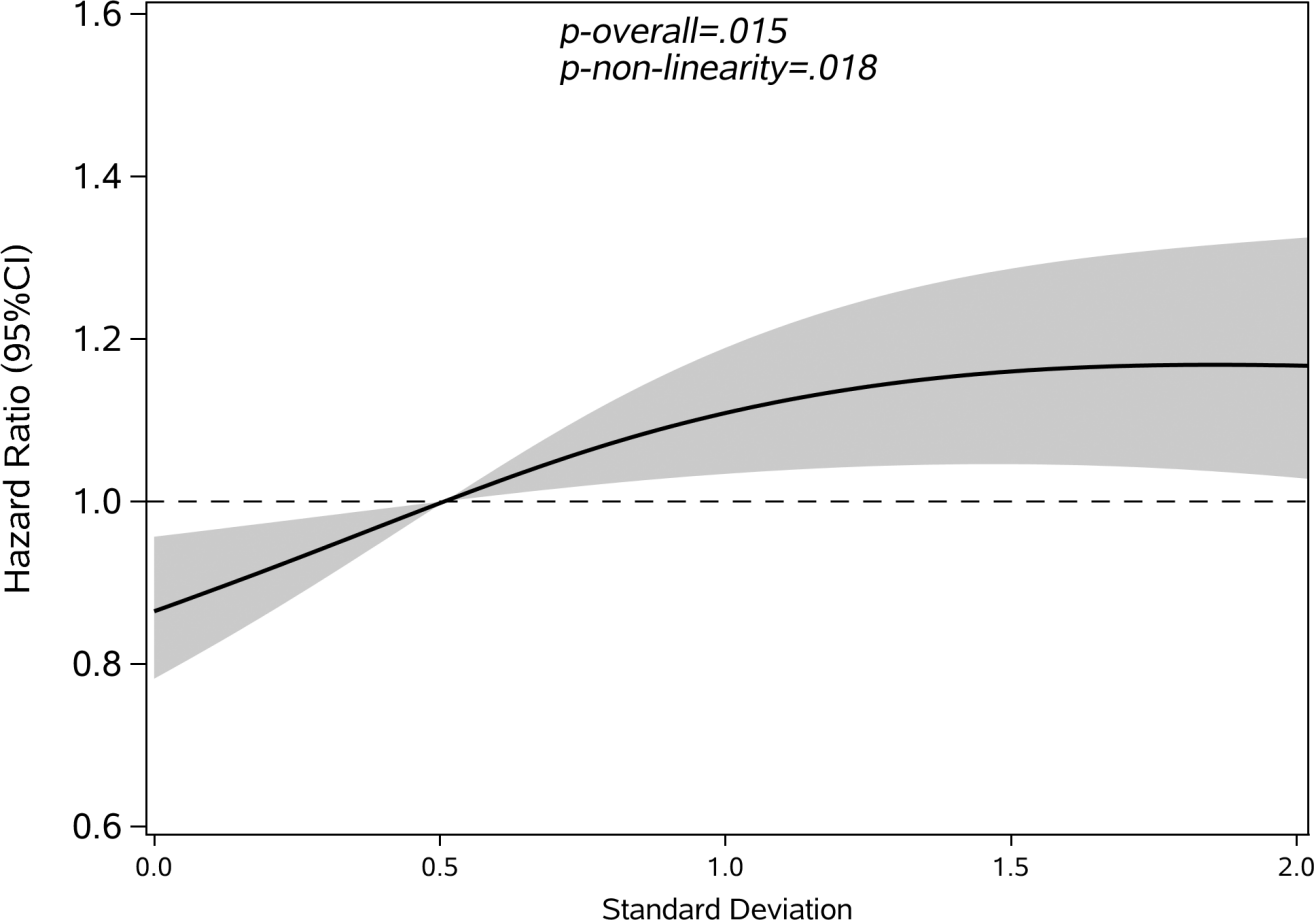
Shape of the association between SD and the development of cancer with type 2 diabetes mellitus (T2DM). The solid lines: hazard ratio; dash areas: 95% confidence interval zone (CI). Data were adjusted for baseline variables, including gender, age, body mass index (BMI), family history of diabetes mellitus (DM), hypertension, hypoglycemic drugs, physical exercise, FBG‐mean, FBG at baseline, follow‐up frequency (Model 3). FBG, fasting blood glucose.

Restricted cubic splines were used to assess and visualize the relationship between FBG variability and cancer. Figures 2, 3, 4, 5 shows the nonlinear dose–response association after adjusting all covariates. For FBG‐VIM, FBG‐ARV, and FBG‐SD, the hazards of cancer began to rise slowly until around the 25th percentile of indicators and leveled off afterward (Figures 2, 4, 5). We observed nonlinearity association of FBG‐VIM (*p* of nonlinear <.001), FBG‐ARV (*p* of nonlinear = .007) and FBG‐SD (*p* of nonlinear = .018) with cancer. Conversely, the cancer risk rose continuously as FBG‐CV increased, and no obvious nonlinearity was found in the dose–response relationship with the continuous changes in FBG‐CV (*p* of nonlinear = .070).

### 3.3 Interaction analysis

The interactive and joint associations of hypertension with FBG variability and hypertension with FBG‐mean on cancer are shown in Table 3. Participants with a high level of FBG‐VIM and hypertension exhibited a lower risk of developing cancer than those with low FBG‐VIM and normotension (HR = 0.67, 95% CI: 0.57, 0.78). The situation is similar for FBG variability evaluated by ARV, CV, and SD, (HR = 0.66, 95% CI: 0.56, 0.77; HR = 0.69, 95% CI: 0.59, 0.80; HR = 0.70, 95% CI: 0.60, 0.81). Participants with hypertension had a conspicuously antagonistic additive effect between FBG‐ARV (RERI: −0.46, 95% CI: −0.76, −0.16), FBG‐CV (RERI: −0.31, 95% CI: −0.58, −0.03), FBG‐SD (RERI: −0.30, 95% CI: −0.57, −0.03) and cancer.

**TABLE 3:** Joint association of average real variability or glucose‐mean and hypertension on cancer risk

Studied variable	Blood pressure category	Case (*N*, %)	Crude incidence (10 000 person‐year)	Cancer risk^a^ (95% CI)	*p*_interaction_
VIM * Hypertension				0.81 (0.66, 0.99)	.040
RERI (95% CI)				−023 (−0.49, 0.04)	.098
Low VIM (mmol/L)	Normotension	200 (3.1)	74.69	1(ref)	
	Hypertension	692 (4.4)	104.51	0.90 (0.79, 1.02)	
High VIM (mmol/L)	Normotension	300 (4.5)	89.29	0.92 (0.83, 1.02)	
	Hypertension	1026 (5.8)	107.72	0.67 (0.57, 0.78)	
ARV * Hypertension				0.68 (0.56, 0.84)	<.001
RERI (95% CI)				−0.46 (−0.76, −0.16)	.002
Low ARV (mmol/L)	Normotension	203 (3.0)	68.87	1 (ref)	
	Hypertension	774 (4.7)	107.71	0.97 (0.85, 1.11)	
High ARV (mmol/L)	Normotension	297 (4.6)	96.07	0.99 (0.89, 1.09)	
	Hypertension	944 (5.6)	105.08	0.66 (0.56, 0.77)	
CV * Hypertension				0.76 (0.63, 0.93)	.009
RERI (95% CI)				−0.31 (−0.58, −0.03)	.027
Low CV (%)	Normotension	215 (3.2)	75.83	1 (ref)	
	Hypertension	783 (4.7)	109.78	0.92 (0.81, 1.06)	
High CV (%)	Normotension	285 (4.4)	88.95	0.97 (0.87, 1.07)	
	Hypertension	935 (5.5)	103.53	0.69 (0.59, 0.80)	
SD * Hypertension				0.77 (0.63, 0.94)	.010
RERI (95%CI)				−0.30 (−0.57, −0.03)	.044
Low SD (mmol/L)	Normotension	219 (3.2)	76.83	1 (ref)	
	Hypertension	806 (4.8)	110.85	0.93 (0.81, 1.06)	
High SD (mmol/L)	Normotension	281 (4.3)	88.08	0.98 (0.88, 1.09)	
	Hypertension	912 (5.4)	102.57	0.70 (0.60, 0.81)	
Mean * Hypertension				0.86 (0.69, 1.07)	.174
RERI (95% CI)				−0.16 (−0.42, 0.08)	.191
Low mean	Normotension	345 (4.2)	81.13	1 (ref)	
	Hypertension	1273 (5.2)	109.64	0.92 (0.76, 1.10)	
High mean	Normotension	155 (3.9)	86.43	1.05 (0.93, 1.18)	
	Hypertension	445 (4.9)	97.63	0.83 (0.71, 0.96)	

^a^ Data were adjusted for baseline variables, including gender, age, BMI, family history of DM, hypertension, hypoglycemic drugs, physical exercise, FBG‐mean, FBG at baseline, and follow‐up frequency.

Abbreviations: ARV, average real variability; BMI, body mass index; CI, confidence interval; CV, coefficient of variation; DM, diabetes mellitus; FBG, fasting blood glucose; Ref, reference; RERI, the relative excess risk because of the interaction; VIM, variation independent of the mean.

However, no noteworthy interaction was observed between FBG‐mean and hypertension (HR = 0.86, 95% CI: 0.69, 1.04; RERI: −0.16, 95% CI: −0.42, 0.08) and hypertension on both multiplicative and additive scale.

Based on these findings, we conducted a subgroup analysis, and the results were consistent with the interaction analysis. For people with hypertension, we found an insignificant correlation between FBG variability and cancer incidence (Table 4).

**TABLE 4:** Association stratified by FBG variability and FBG‐mean

Studied factor	Cancer risk (95% CI)^a^	
	Blood pressure category	
VIM (mmol/L)	Normotension	Hypertension
≤0.16	1 (ref)	1 (ref)
>0.16	1.36 (1.13, 1.65)	1.09 (0.99, 1.20)
ARV (mmol/L)		
≤0.40	1 (ref)	1 (ref)
>0.40	1.53 (1.26, 1.86)	1.00 (0.90, 1.10)
CV (%)		
≤7.80	1 (ref)	1 (ref)
>7.80	1.38 (1.13, 1.68)	1.02 (0.92, 1.13)
SD (mmol/L)		
≤0.51	1 (ref)	1 (ref)
>0.51	1.37 (1.12, 1.68)	1.01 (0.91, 1.12)
Mean (mmol/L)		
≤7.20	1 (ref)	1 (ref)
>7.20	1.15 (0.93, 1.42)	0.94 (0.84, 1.06)

^a^ Data were adjusted for baseline variables, including gender, age, BMI, family history of DM, hypertension, hypoglycemic drugs, physical exercise, FBG‐mean, FBG at baseline, and follow‐up frequency.

Abbreviations: ARV, average real variability; BMI, body mass index; CI, confidence interval; CV, coefficient of variation; DM, diabetes mellitus; FBG, fasting blood glucose; Ref, reference; VIM, variation independent of the mean.

## 4 DISCUSSION

In this retrospective cohort study of 46 761 individuals in the Minhang area, Shanghai, we reported a positive association between long‐term FBG‐VIM, FBG‐ARV, FBG‐CV, and FBG‐SD and the risk of cancer. The association was independent of age, gender, weight, height, BMI, hypoglycemic drug use, family history of diabetes, daily exercise, frequency of follow‐up, comorbidity of hypertension, FBG‐mean, and FBG at baseline. Dose‐response association analysis demonstrated a nonlinearly increased connection strength of cancer with the continuous changes in FBG. Finally, we discovered antagonistic interactive effects between FBG variability and hypertension on the onset of cancer.

Multiple population‐based studies have documented the impact of FBG variability on cancer.^25^ Measuring the long‐term variability of fasting plasma glucose was proved to be useful for predicting the risk for cancer mortality in a Chinese study.^11^ According to a Hong Kong diabetes register cohort study, per SD increase of glycemic variability was related to an adjusted HR of 1.21 (95% CI: 1.06, 1.39) for all‐sites cancer death in the median high glycemic variability group. These previous studies primarily concentrated on cancer mortality rather than incidence.^26^ Furthermore, a retrospective cohort study observed a dose‐dependent link between glycemic variability and tumorigenesis, with an odds ratio of 2.19 (95% CI: 1.52, 3.17) for the fourth quartile of glycemic variability. However, they enrolled only a rather limited sample of patients with DM.^12^ A Korean study confirmed 5494 cases of hepatocellular cancer during a median follow‐up of 6.7 years and found that the risk rose by 27% for the highest FBG variability compared to the lowest quartile.^13^ Another study from the same cohort reported that FBG variability was significantly associated with a 10% increased risk of gastric cancer. These studies focused on specific‐site or ‐organ cancers but not all‐sites cancer. Results slightly differed among previous studies due to different selection criteria and inclusion of covariates. Our research supported the positive association between FBG variability and all‐sites cancer among T2DM patients.^19^ The findings on FBG variability were in accordance with those from previous research that observed a nonlinear association with cancer incidence grouped by gender. Furthermore, we added a new viewpoint that the observed nonlinear association remained even though we use different variability indicators regardless of gender.

Several possible pathophysiological pathways could account for the association between long‐term FBG variability and elevated risk of cancer in T2DM patients.^27^ First, cancer initiation was closely linked to oxidative stress and chronic tissue inflammation.^28^ FBG variability tended to aggravate oxidative stress and impose chronic damage on beta‐cell function, including overexpression of oxidative biomarkers, excessive generation of free radical oxygen (ROS) species and cellular apoptosis.^29^ It also enhanced free radical formation and activation of the poly (adenosine diphosphate‐ribose) polymerases (PARP) pathway^30^ and promoted vascular endothelial senescence.^31^ A small population‐based study found that both acute and chronic FBG variability could produce ROS that causes oxidative stress, chronic inflammation, somatic mutations, and neoplastic transformation. Our study further highlighted the effect of FBG variability even when accounting for FBG‐mean, because the result became significant after adjusting for FBG‐mean and it may work greater than FBG‐mean in the physiological process above.^32^ In addition, FBG‐variability‐related genes were associated with insulin resistance and hyperinsulinemia.^33^ Insulin receptor A isoform may overexpress in cancer cells to provide a selective growth advantage to malignant cells exposed to hyperinsulinemia.

Another possible explanation for the positive association between FBG variability and cancer is that^34^ BMI,^35^ age,^36^ and hypoglycemic drug use may influence the link chain. Thus, we adjusted models for these factors.^37^ Cancer can induce altered glucose metabolism. To preclude possible reverse causation, we excluded participants who had been diagnosed with cancer within 6 months of registering the cohort.

Our results showed that in the T2DM population, FBG variability and hypertension had a significant negative interaction effect on cancer risk, whereas FBG‐mean showed an insignificant effect. The reasons for the interaction of FBG variability with hypertension on risks of cancer are uncertain. One possible hypothesis suggested that the results may be biased by confounding factors, such as diet conditions and drug type of hypertension, which played important roles in the pathways of the interactive effects. To be specific, as an effective treatment to reduce blood pressure, Dietary Approach to Stop Hypertension (DASH) diet may improve glycemic control according to a randomized controlled trial. Angiotensin converting enzyme inhibitors/angiotensin II receptor blockers, one of the antihypertensive drugs,^38^ affected IR protectively^39^ and therefore may increase cancer risk. Moreover, hypertensive participants may stick to healthy lifestyles compared with nonhypertensive ones. Limited by the availability of data, our study did not include these potential confounders. The other possible reason was mutual antagonism.^40^ The coexistence of hypertension and FBG variability may interfere with the effect of respective exposure though they caused the onset of cancer individually. Antagonistic additive interaction seemingly provides evidence to implement public health interventions in high‐risk population such as hypertensive patients.

One strength of our study was the reliable and extensive data from large public health programs. We further interpreted FBG variability by four different indices, which enabled us to evaluate longitudinal FBG variability practically.^41^ VIM reflects the distribution of FBG within the cohort and much fewer dependents on FBG‐mean.^42^ ARV is a more reliable representation of time series variability.^43^ We could better quantify the FBG variability and eradicate the influence of FBG‐mean. Consistent results calculated by various indexes indicated that long‐term changes in FBG could increase the hazards of cancer incidence. Also, we applied a restricted cubic spline function to demonstrate the dose–response association more accurately and avoid the loss of information when translating the FBG variability into a categorical variable.

There are several limitations to our study.^44^ First, owing to the lack of information, we did not use data on hemoglobin A1c (HbA_1c_) variability that may be strongly associated with cancer.^45^ FBG variability may reflect real glycemic change better than HbA_1c_ levels because HbA_1c_ levels could be affected by nonglycemic factors. Second, some unknown and complicated confounders may remain, which are likely to misestimate the actual association. Some variables were self‐reported and they may result in subjectivity bias.^46^ In addition, FBG variability is influenced by the number of visits used to calculate it. Although we did not calculate the indicators with the same frequency of visits, we adjusted for it in regression analysis to minimize its impact.^18^ This method of investigation has proved to be practical and effective in the previous report using the same questionnaire. Finally, because this study was not an intervention or prospective study but a retrospective cohort analysis, causality cannot be determined. In general, additional studies on the association of FBG variability and cancer that include comprehensive measurements and objective covariates are warranted.

## 5 CONCLUSION

In our research, high FBG variability was positively associated with increased hazards of all‐sites cancer. We found a nonlinear and linear link between FBG variability and cancer when measurements differ. Hence, long‐term changes in FBG variability may be used to identify patients with T2DM who are at high cancer risk and should be targeted for treatment to avoid cancer. We found a negative interaction between FBG variability and comorbidity of hypertension on cancer risk.

## FUNDING INFORMATION

The authors thank all the staff and the participants in the project. This research was supported by the National Natural Science Foundation of China (grants 82173612 and 82273730); Shanghai Rising‐Star Program (21QA1401300); Shanghai Municipal Natural Science Foundation (22ZR1414900); National Key R&D Program of China (2021YFC2502200); Shanghai Municipal Science and Technology Major Project (ZD2021CY001).

## DISCLOSURE

No potential conflicts of interest relevant to this article were disclosed.
